# Supplementary material for: Disparities in Cancer Incidence across Income Levels in South Korea
Source: Cancers (Basel). 2023 Dec 18;15(24):5898. doi: 10.3390/cancers15245898 (PMC10741676; doi:10.3390/cancers15245898)
Supplement: Supplementary file 1 [file cancers-15-05898-s001.zip › cancers-2752066-supplementary.pdf]

Supplementary Table S1. Number of cancer patients by cancer type and income level (total).

	No. of population	Cancer patients (all cancer)	Gastric	Colorectal	Liver	Lung	Thyroid	Breast (Female)	Cervical (Female)	Prostate (Male)
Medical aid	1,484,671	11,226	1,314	1,589	1,118	1,690	565	742	166	540
1Q (lowest)	7,056,101	34,849	4,261	4,321	2,373	3,847	4,102	4,026	676	1,764
2Q	7,251,612	30,181	3,638	3,670	2,121	3,213	3,977	3,375	596	1,546
3Q	8,747,805	35,655	4,388	4,366	2,435	3,704	4,867	3,689	645	1,873
4Q	10,819,897	45,540	5,607	5,237	2,806	4,830	6,454	4,557	690	2,705
5Q (highest)	13,829,966	65,920	8,157	7,179	3,727	7,395	8,110	6,243	672	5,103

Supplementary Table S2. Number of cancer patients by cancer type and income level (male).

	No. of population	Cancer patients (all cancer)	Gastric	Colorectal	Liver	Lung	Thyroid	Prostate
Medical aid	676,449	5,888	806	877	776	1,093	57	540
1Q (lowest)	3,159,927	16,637	2,737	2,493	1,763	2,539	606	1,764
2Q	3,475,205	15,319	2,544	2,304	1,623	2,238	624	1,546
3Q	4,482,162	18,601	3,074	2,679	1,920	2,550	1,042	1,873
4Q	5,698,639	24,298	3,929	3,264	2,153	3,360	1,747	2,705
5Q (highest)	7,126,302	35,577	5,545	4,091	2,677	4,975	2,530	5,103

Supplementary Table S3. Number of cancer patients by cancer type and income level (female).

	No. of population	Cancer patients (all cancer)	Gastric	Colorectal	Liver	Lung	Thyroid	Breast	Cervical
Medical aid	808,222	5,338	508	712	342	597	508	742	166
1Q (lowest)	3,896,174	18,212	1,524	1,828	610	1,308	3,496	4,026	676
2Q	3,776,407	14,862	1,094	1,366	498	975	3,353	3,375	596
3Q	4,265,643	17,054	1,314	1,687	515	1,154	3,825	3,689	645
4Q	5,121,258	21,242	1,678	1,973	653	1,470	4,707	4,557	690
5Q (highest)	6,703,664	30,343	2,612	3,088	1,050	2,420	5,580	6,243	672

Supplementary Table S4. Slope Index of Inequality (SII) and Relative Index of Inequality (RII) of age-standardized incidence rate (per 100,000 population): male.

Cancer Type	Medical Aid	Health Insurance Subscribers					SII	SII 95% CI		RII	RII 95% CI	
		1Q	2Q	3Q	4Q	5Q						
All cancer	594.4	542.6	583.4	536.1	553.8	527.6	-43.24	-120.22	33.74	-0.08	-0.22	0.06
Stomach	80.8	87.3	91.2	87.2	88.8	81.9	-7.36	-20.61	5.89	-0.09	-0.24	0.07
Colorectal	87.2	80.1	84.8	76.0	74.5	60.7	**-29.11**	**-44.88**	**-13.34**	**-0.38**	**-0.58**	**-0.17**
Liver	81.8	56.5	57.2	52.5	48.3	39.8	**-27.48**	**-44.89**	**-10.06**	**-0.49**	**-0.80**	**-0.18**
Lung	102.1	85.3	97.6	82.1	84.5	73.5	-23.50	-47.46	0.46	-0.27	-0.54	0.01
Thyroid	6.8	19.1	17.2	21.5	28.9	37.8	**29.31**	**18.85**	**39.78**	**1.34**	**0.86**	**1.82**
Prostate	49.4	59.4	67.6	60.4	67.1	76.8	**22.11**	**4.46**	**39.75**	**0.35**	**0.07**	**0.63**

Supplementary Table S5. Slope Index of Inequality (SII) and Relative Index of Inequality (RII) of age-standardized incidence rate (per 100,000 population): female.

**Cancer Type**	**Medical Aid**	**Health Insurance Subscribers**					**SII**	**SII 95% CI**	**RII**	**RII 95% CI**
		**1Q**	**2Q**	**3Q**	**4Q**	**5Q**				
All cancer	440.5	425.9	429.5	414.7	436.6	421.5	-4.89	-42.31 32.53	-0.01	-0.10 0.08
Stomach	32.6	35.1	34.7	33.3	35.1	32.9	-1.79	-6.02 2.44	-0.05	-0.18 0.07
Colorectal	45.3	41.8	44.3	43.6	41.5	37.4	**-7.64**	**-15.19 -0.10**	-0.18	-0.36 0.00
Liver	24.6	13.9	17.5	13.5	13.7	12.0	-6.11	-15.59 3.38	-0.38	-0.98 0.21
Lung	34.9	29.8	33.4	30.4	30.9	29.9	-2.23	-8.44 3.98	-0.07	-0.27 0.13
Thyroid	73.3	85.1	81.9	84.7	93.2	93.2	**16.51**	**3.22 29.80**	**0.19**	**0.04 0.35**
Breast (Female)	87.4	94.3	87.8	87.5	93.7	95.0	5.96	-7.93 19.84	0.07	-0.09 0.22
Cervical	18.2	16.1	15.4	15.0	13.8	10.4	**-7.77**	**-11.09 -4.44**	**-0.52**	**-0.75 -0.30**
